# Multifocal and recurrent cutaneous pseudolymphoma associated with lamotrigine and review of the literature

**DOI:** 10.1016/j.jdcr.2022.07.040

**Published:** 2022-08-10

**Authors:** Soroush Kazemi, Elanee Simmons, Maija Kiuru, Danielle M. Tartar

**Affiliations:** Department of Dermatology, University of California Davis, Sacramento, California

**Keywords:** drug reaction, lamotrigine, pseudolymphoma, CPL, cutaneous pseudolymphoma

## Introduction

Cutaneous pseudolymphoma (CPL) is a benign lymphoproliferative condition that clinically mimics malignant cutaneous lymphoma. CPL often presents as a solitary violaceous–erythematous papule, nodule, or plaque. Rare multifocal or disseminated cases have been reported.^1^ While most cases of CPL are idiopathic, known triggers include photosensitivity, HIV, Varicella zoster virus, *Borrelia burgdorferi*, tattoos, minor trauma, insect bites, and medications.^1^ Some of the medication classes known to cause drug-induced CPL are anticonvulsants, tumor necrosis factor-alpha inhibitors, antibiotics, and antihypertensives. Among these drugs, phenytoin and carbamazepine are the most common culprits. Herein, we report a case of multifocal, recurrent CPL induced by lamotrigine, which is a triazine anticonvulsant medication.

## Case report

**Fig 1:**
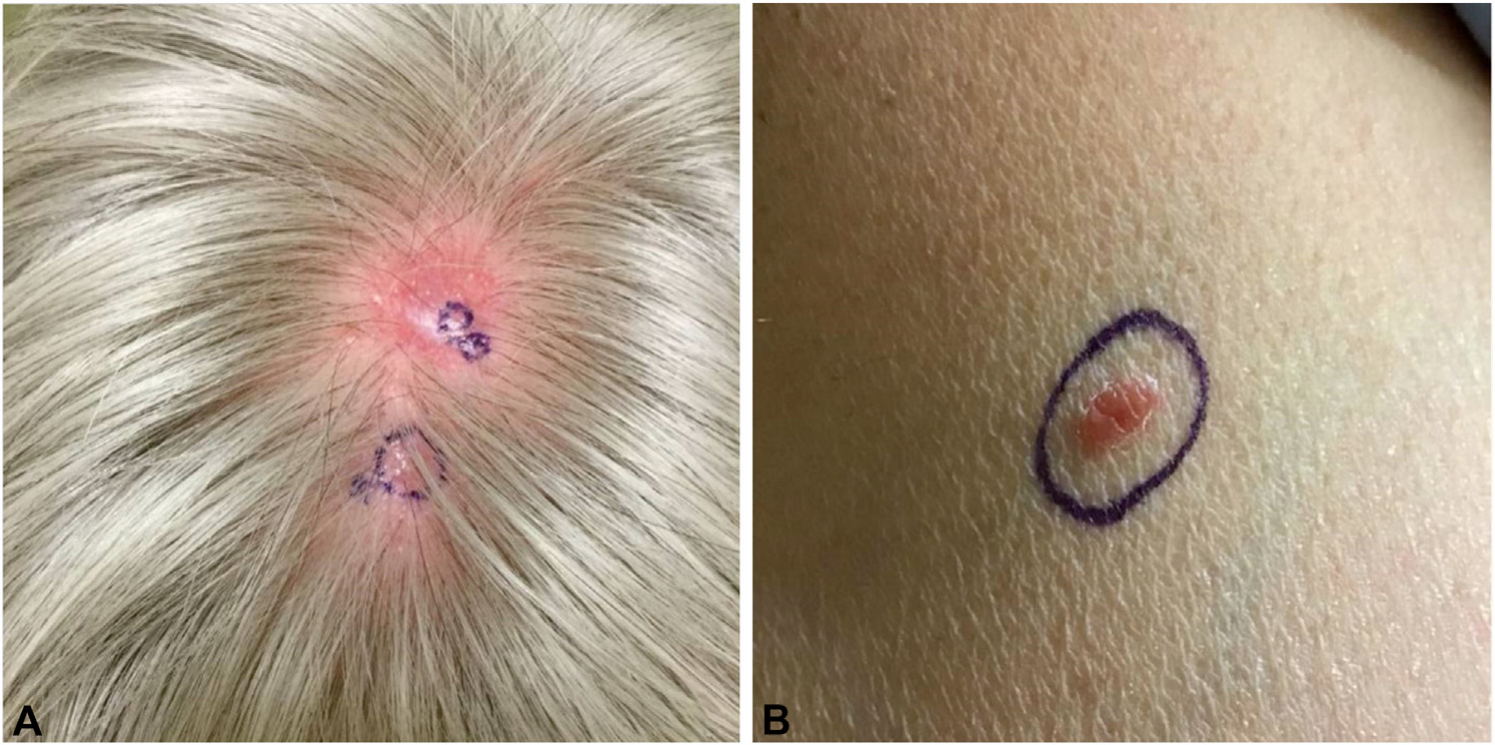
**A,***Red* pruritic papules on vertex scalp upon initial presentation; (**B**) new papule on the right chest 4 months after initial presentation.

A 59-year-old Caucasian female with a history significant for bipolar 2 disorder presented to dermatology with new onset red, pruritic papules on her vertex scalp with associated alopecia (Fig 1, *A*).

The family history was significant for unknown lymphoma in her father, which resulted in death. She had no lymphadenopathy or constitutional symptoms. She denied prior trauma or injury to the affected areas. Medications were significant for lamotrigine 150 mg daily, escitalopram 10 mg daily, methylphenidate 15 mg twice daily, and clonazepam 1.5 mg daily.

**Fig 2:**
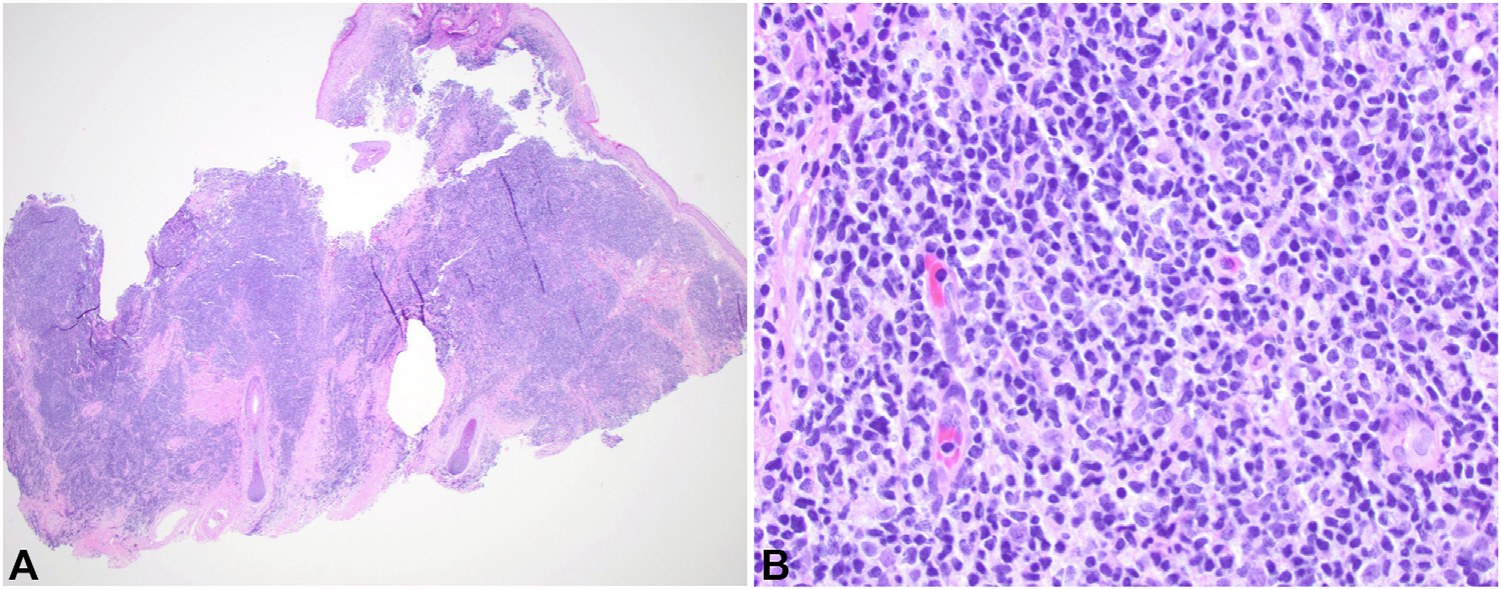
Hematoxylin and eosin revealed a dense nodular and interstitial infiltrate composed of lymphoid cells throughout the dermis (**A**: 2×, **B**: 40×).

Initial biopsy revealed an atypical mixed lymphocytic infiltrate (Fig 2). Immunohistochemical staining was positive for both CD3 and CD20, and there was no evidence of light chain restriction by kappa or lambda immunohistochemistry. There was predominance of CD4 over CD8 T cells, and CD30 was negative. B-cell and T-cell gene rearrangement studies performed on 2 separate specimens returned negative. Complete blood count, differentials, serum chemistries, lactase dehydrogenase, and serum CD4/CD8 ratio were within normal limits. The patient was diagnosed with CPL of unknown etiology and started on topical hydrocortisone 1% cream with no improvement. She developed 5 new papules on her scalp as well as a new nodule on her right chest (Fig 1, *B*).

Additional biopsy of the right chest revealed similar findings with a similar immunohistochemical profile. Intralesional triamcinolone (40 mg/mL) was injected into individual lesions with improvement, though she continued to grow new lesions, both de novo and at the site of prior injections. Four total sites were biopsied, all with similar histopathologic findings. Given the recurrent and ongoing nature, oncology was consulted and positron emission tomography/computed tomography was completed, which showed no evidence of systemic disease or lymphoma. Out of concern for lamotrigine-induced CPL, lamotrigine was weaned over a 3-weeks period with the help of the patient’s psychiatrist, with complete resolution of the lesions within 1 one month. One-, 2-, and 3-month follow-up with the patient showed no lesions. A diagnosis of lamotrigine-induced CPL was made. Of note, the patient was maintained on methylphenidate, which has also been reported in association with CPL (Table I).

**Table I:** Nonanticonvulsant drugs causing pseudolymphoma with cutaneous presentations

Drug class	Number of cases reported	Specific drug	Presentation
Immunosuppressants	12	Azathioprine (3), Cyclosporine (3), Tacrolimus (2), Methotrexate (2) Mycophenolate mofetil (1), Tocilizumab (1)	Solitary cutaneous nodule^2^; solitary visceral ulcer with cutaneous invasion^2^; multifocal cutaneous nodules^3^^,^^4^; localized cutaneous nodules with localized lymphadenopathy^2^; generalized lymphadenopathy with generalized rash^5^
Tumor necrosis factor-alpha inhibitors	4	Infliximab (2), Etanercept (1), Adalimumab (1)	Solitary cutaneous ulcer^6^; multifocal cutaneous nodules^7^^,^^8^
Antibiotics	2	Vancomycin (1) Levofloxacin (1)	Generalized cutaneous nodules^9^^,^^10^
Antihypertensives	2	Atenolol (1) ACE inhibitor (1)	Generalized lymphadenopathy with generalized rash^10^, ^11^
Bisphosphonates	1	Zoledronic acid (1)	Localized cutaneous nodules^12^, ^13^
Stimulants	1	Methylphenidate (1)	Multifocal cutaneous nodules^10^
Antidepressents	1	Fluoxetine (1) Amitriptyline (1)	Localized cutaneous nodules^10^, ^14^, ^15^
Other	1	Bromocriptine (1)	Multifocal nodules^16^

## Discussion

Anticonvulsant medications are the most commonly associated medications with drug-induced CPL. A review of anticonvulsant-induced pseudolymphoma is summarized in Table II. Only reports containing cutaneous findings were included.

**Table II:** Different anticonvulsants causing pseudolymphoma with cutaneous presentations

Anticonvulsant	Number of cases reported	Presentation
Lamotrigine	2	Localized cutaneous nodules^17^; generalized cutaneous nodules^18^
Carbamazepine	12	Solitary cutaneous nodule^19^^,^^20^; localized cutaneous nodules^21^^,^^22^; generalized cutaneous nodules^23^, ^24^, ^25^, ^26^; generalized lymphadenopathy with generalized rash^27^, ^28^, ^29^, ^30^
Phenytoin	9	Localized cutaneous nodules^31^; generalized cutaneous nodules^32^^,^^33^; multifocal cutaneous plaques^34^; generalized cutaneous plaques^35^^,^^36^; generalized lymphadenopathy with generalized rash^37^, ^38^, ^39^
Gabapentin	2	Solitary cutaneous plaque^40^; localized cutaneous nodules^41^
Valproate	1	Solitary cutaneous nodule^21^

There are limited case reports involving lamotrigine compared to phenytoin and carbamazepine, and these 2 drugs have also been shown to cause CPL that can mimic mycosis fungoides.^23^, ^24^, ^34^, ^37^, ^42^ Other medication classes reported to cause pseudolymphoma, cutaneous lymphoid hyperplasia, or lymphocytoma cutis (alternative names for the same phenomenon) in our review of the literature are summarized in Table I. As with Table II, only reports containing cutaneous findings were included.

This patient’s clinical and histopathologic findings were concerning for cutaneous lymphoma as well as primary cutaneous CD4+ small/medium T-cell lymphoproliferative disorder. The negative workup made cutaneous lymphoma less likely. Based on the histology alone, we could not definitively differentiate between CPL versus primary cutaneous CD4+ small/medium T-cell lymphoproliferative disorder, but the resolution of the cutaneous lesions after discontinuation of lamotrigine was more consistent with multifocal, recurrent CPL. It is unclear if the number and distribution of lesions are indicative of drug-induced CPL versus CPL of other etiology, and further study is required.

CPL is generally regarded as benign, but there is still a risk of malignant transformation in the future. This condition is known as pseudo-pseudolymphoma, and 1 study showed 4% of patients with CPL progressed to a true malignant lymphoma.^43^ While our report had complete resolution after discontinuation of lamotrigine, it is important to stay vigilant and follow up with CPL patients in the rare chance that they develop a malignancy. This case highlights the need to consider a drug-induced syndrome. Further case reports of lamotrigine-induced CPL are needed to greater understand this phenomenon and assess for the incidence of true malignant lymphoma in these patients.

## Conflicts of interest

None disclosed.
